# Harnessing Digital Health Technologies During and After the COVID-19 Pandemic: Context Matters

**DOI:** 10.2196/21815

**Published:** 2020-12-30

**Authors:** Francesco Petracca, Oriana Ciani, Maria Cucciniello, Rosanna Tarricone

**Affiliations:** 1 Centre for Research in Health and Social Care Management (CERGAS) Government, Health and Non Profit Division SDA Bocconi Milan Italy; 2 Institute of Health Research University of Exeter Medical School Exeter United Kingdom; 3 University of Edinburgh Business School Edinburgh United Kingdom; 4 Department of Social and Political Sciences Bocconi University Milan Italy

**Keywords:** mobile apps, coronavirus, COVID-19, digital health, mHealth, organizational context, public health, telemedicine

## Abstract

A common development observed during the COVID-19 pandemic is the renewed reliance on digital health technologies. Prior to the pandemic, the uptake of digital health technologies to directly strengthen public health systems had been unsatisfactory; however, a relentless acceleration took place within health care systems during the COVID-19 pandemic. Therefore, digital health technologies could not be prescinded from the organizational and institutional merits of the systems in which they were introduced. The Italian National Health Service is strongly decentralized, with the national government exercising general stewardship and regions responsible for the delivery of health care services. Together with the substantial lack of digital efforts previously, these institutional characteristics resulted in delays in the uptake of appropriate solutions, territorial differences, and issues in engaging the appropriate health care professionals during the pandemic. An in-depth analysis of the organizational context is instrumental in fully interpreting the contribution of digital health during the pandemic and providing the foundation for the digital reconstruction of what is to come after.

## The Unparalleled Surge in Digital Health Adoption

The COVID-19 pandemic has presented governments, managers, and professionals worldwide with unprecedented challenges, highlighting the limitations of analog health care systems that have long reinforced face-to-face models of care and congregation of individuals [1]. Regardless of the diverse containment and mitigation strategies implemented across countries, a common development observed during the pandemic is the renewed reliance on digital health, with efficient strategies implemented at various levels and directed at different stakeholders [2,3].

Rapid implementation of social distancing measures and rescheduling of elective procedures have led health care providers to resort to digital health applications for granting access to virtual consultations and remote visits and monitoring [4]. In short, health care has come to patients rather than the other way round.

As a result, during the first 3 months of the pandemic, an unparalleled surge in digital health adoption was observed, with a general scale-up of telemedicine [5] and a substantial shift to telehealth visits in some clinics [6], as confirmed by the increased number of telehealth services and e-consultations reported in several jurisdictions [6-8].

The fact that these technologies were available and ready for use should not have come as a surprise. However, before the COVID-19 outbreak, the uptake of digital health technologies to strengthen public health systems had been unsatisfactory, and the scholarly debate had rather focused on the barriers to adoption of digital health solutions [9,10] and difficulties in governing mobile apps [11]. Nonetheless, the adoption of digital health solutions, regardless of whether it occurred during or after the pandemic, cannot be prescinded from the organizational and institutional merits of the systems in which they are introduced. The full potential of technological innovation can be realized only by way of heightened attention to context.

## COVID-19 Digital Health Experience in the Italian National Health Service

Italy was among the first and most significantly hit countries by SARS-CoV-2. As of July 21, 2020, Italy reported a total of 244,708 confirmed cases, over 34,000 COVID-19–related deaths, and the highest deaths per million inhabitants and case fatality rates worldwide [12-14].

In terms of institutional composition, the Italian National Health Service (SSN) is strongly decentralized and based on universal access to care. The central government exercises general stewardship, whereas 21 regions—each considerably different in size and economic development and autonomous in their health care management decisions—are responsible for the organization and delivery of primary, secondary, tertiary, and preventive health care services through local health authorities (LHAs) [15].

Before the COVID-19 emergency, adoption of digital health was a challenge in Italy, as elsewhere. By analyzing the distinctive digital health experiences during the first wave of the pandemic in Italy—a country that was on the front line of the pandemic [16,17], we aimed to showcase the best practices, open questions, and barriers encountered. Systems, institutions, and organizations who have attempted to embrace digital health in the past months and are looking to maintain the momentum after COVID-19 have experienced similar challenges. Some of these challenges are related to issues such as reimbursement of digital health services, experience of using contact tracing apps, the search for balance between local experiences and national stewardship, arduous involvement of all health care professionals, and the coexistence of digital and analog pathways.

### Reimbursement Schemes

Appropriate reimbursement schemes are essential to expanding the role of digital technologies. During the current public health crisis, telemedicine services promptly seemed to be vital to support remote monitoring of less severe cases and ensure continuity of care for vulnerable, typically chronic patients, whose needs were overshadowed by the surge in patients with COVID-19 seeking assistance. However, telemedicine services in Italy have traditionally been scattered across a number of different applications, with poor interconnection and inconsistent local and regional reimbursement practices, since they are not specifically covered by the guaranteed health basket of the Italian SSN [18]. No extensive, nationwide input was provided by health authorities during the pandemic, as has been the case of France, where the Ministry of Health allowed the reimbursement of teleconsultations for suspected and confirmed COVID-19 cases [6]. The Italian National Institute of Health (Istituto Superiore di Sanità) proposed a temporary model to ease the implementation of telemedicine services during the emergency, but this essentially focused on the organizational requirements for telemedicine services and did not tackle the reimbursement domain [19]. The delay in the adoption of national guidance and specific reimbursement codes for telemedicine services resulted in inter-regional differences: jurisdictions such as Veneto, the Autonomous Province of Trento, and Tuscany promptly introduced digital health services as part of their discretionary guaranteed health benefits packages, and were followed by other pioneering regions in the following months. These packages are now being managed online with dedicated tariffs for the citizens of the above regions. Conversely, the remaining regions could only fund telemedicine services by implicitly allowing payment parity with standard outpatient procedures through regional discretionary spending. While the lack of a framework to pay for telehealth services hindered wider-scale adoption by public institutions, private telemonitoring service providers reported a marked increase in the use of direct-to-consumer services [18].

### Contact Tracing Apps

Use of contact tracing techniques and apps during the pandemic exposed the challenges of safeguarding privacy in the development of prompt digital responses to address current and future needs. National contact tracing apps were identified as a key tool to tackle the epidemic and facilitate reopening of the economy. However, unsurprisingly, the design and adoption of these applications, along with associated evaluation timelines, have raised significant privacy and security concerns and resulted in considerable delays in their deployment during the pandemic. Like most European countries, Italy initially backed the adoption of centralized management and maintenance of contact tracing data, including the “social graph” of individuals a person has physically met over a given period [20]. This practice was in contrast with the view of the European Commission, which supported anonymous, aggregated, and decentralized approaches to contact tracing to preserve privacy [21]. Meanwhile, Apple and Google had collaborated in a novel partnership to develop a safe infrastructure and privacy protection standards to specifically tackle these operations [22]. However, voluntary, data minimization systems are considered the sole option to build acceptance and maximize the uptake of these solutions [23]. These systems preserve user anonymity while providing useful warnings to other users who may have recently come into close physical proximity of a certain individual who subsequently tested positive for COVID-19. Eventually, together with several other European countries [24], the Italian government shifted focus towards these types of Bluetooth Low Energy apps that interfere as little as possible with users’ privacy rights.

Collectively, lengthy negotiations on privacy standards and relatively scarce previous attempts to evaluate these technologies have delayed the widespread roll-out of functional contact-tracing apps.

Immuni, an app selected by an adhoc task force of the Italian Ministry of Health [25], was launched nationwide on June 15, 2020, that is, 6 weeks after the first lockdown in Italy was lifted (May 4, 2020). Despite significant setbacks that delayed the app deployment, Italy was still among the first European countries to roll-out a contact-tracing solution, as proof of the broad-based intricacy of this matter. Nonetheless, the number of app downloads (4.3 million) for Immuni has been disappointingly low so far and is insufficient to guarantee the effectiveness of the tool [26].

### Balancing Local Experiences and National Responsibilities

Leveraging local experiences with digital health projects in response to the crisis must be balanced with the national governments’ global policy-setting responsibilities. Highly regionalized health systems, such as the Italian SSN, can generate both significant opportunities for innovation as well as threats to national public health initiatives. Moreover, territorial differences in digital response to the COVID-19 crisis that have emerged could provide a basis for mutual learning. For instance, some regional health care systems were better prepared than others, not just in terms of telemedicine policies. In addition, in territories where large-scale digital health projects were ongoing, LHAs were able to rapidly apply them to the needs of the COVID-19 pandemic. For example, in 2015, the LHA of the Autonomous Province of Trento adopted TreC, a patient monitoring system that includes a mobile diary app and a web-based dashboard to facilitate patient-physician interaction and reduce direct access to hospitals. This system was designed to be interoperable with various electronic health records and was tested in several chronic populations [27-29]. After the COVID-19 outbreak in February 2020, the LHA, together with the research entity Fondazione Bruno Kessler, launched TreCovid19—an app built on the same infrastructure designed to inform citizens and enforce remote symptom monitoring. By the end of the pandemic peak, more than 9400 infected or quarantined cases from the province of Trento were actively monitored through this platform, with over 40,000 single parameters directly reported via the chatbot and approximately 700,000 individuals' information accessed via personal interviews with a local executive (data not reported elsewhere).

In the Puglia region in Southern Italy, similar work was being carried out for years on remote follow-up management of chronic patients through several pilot projects. This enabled the implementation of a remote monitoring system based on a web-based application for citizens, a web portal for health care professionals, and a telemedicine kit. The platform, called #Accasa, was implemented on April 22, 2020, for individuals under quarantine or those who have tested positive for COVID-19 [30]. Conversely, boundaries are sometimes blurred in decentralized systems when close cooperation among different institutional levels is required. Several related apps have proliferated in the market during the COVID-19 pandemic, often without the necessary interoperability described above and with overlapping or even hindering features concerning their proposed nationwide application. Although the central government was working on selecting and deploying the most appropriate solutions, all Italian regional systems have worked on their own apps. Initially, these apps only provided information to local communities; however, over time, extra functionalities were added, some of which were slated for inclusion in the design of the national contact-tracing app. For example, Friuli–Venezia Giulia in Northeast Italy had successfully completed the testing of the same app used for contact tracing in Singapore [31], whereas the island of Sardinia had actively sought solutions to track tourists in view of the upcoming summer holiday season. Both attempts were set aside, as contact-tracing needed to be managed nationally. Given that there was no guarantee that the regional apps would be interoperable with the national app, it was fundamental, but not trivial, to ensure that widespread adoption of the national contact-tracing app was not hampered by local and regional initiatives that had been in use for weeks before the national app was launched.

### Involvement of Health Care Professionals

Designing a successful digital health solution requires advanced alignment of the interests of all health care professionals and stakeholders involved. Management of the COVID-19 pandemic is challenging for the health care systems in many ways, including maintaining a balance and relative responsibilities of hospital vis-à-vis community care. It has become clear over time that primary care professionals should play a central role in managing less severe COVID-19 cases via home-based monitoring, possibly by using digital solutions. However, in Italy, general practitioners are contracted professionals. Therefore, it has been much more complex to extend institutionally sponsored digital tools to these third parties and guarantee the necessary system interoperability between services deployed in primary care clinics and all others provided by LHAs. Elsewhere, however, this has been pivotal in implementing a successful digital strategy during the pandemic [7].

### Coexistence of Digital and Analog Pathways for Health Care

The analog approach is not going away any time soon, so analog and digital health systems will need to coexist. Although digital health applications have spread in parallel to the virus, they have rarely been proposed as the only available option. None of the digital solutions implemented in the Italian SSN have been imposed as a mandatory alternative; rather, they have been added to the mix of traditional in-person and telephone-based services to maximize coverage for the entire population. Despite the increased adoption of smartphone use, Italy’s overall population and health care workforce are among the “oldest” in the world [32]. The digital divide is still an issue, and COVID-19 has taught us that digital health is an important option to have in place, but it is no panacea.

## Building on the Momentum

Despite the unprecedented expansion in the utilization of digital health tools during the COVID-19 pandemic, Italy, like many other countries worldwide, was not sufficiently equipped to harness the full potential of these tools. The urgency of the current public health emergency may have set the grounds for making exceptions [33], but the acceleration could not bypass the institutional and organizational elements that characterize national health care systems.

If we intend to maintain the momentum in expanding the digital health services that have resulted from the COVID-19 pandemic, now is the time to effectively plan for the future and to follow a new path, distinct from the pre-pandemic models [33]. The path must be selected by paying particular attention to avoid disparities in access to care. In theory, telemedicine can mitigate this issue but, if not carefully planned, it could also exacerbate it [34].

### Achieving Sustained Benefits

**Figure 1:**
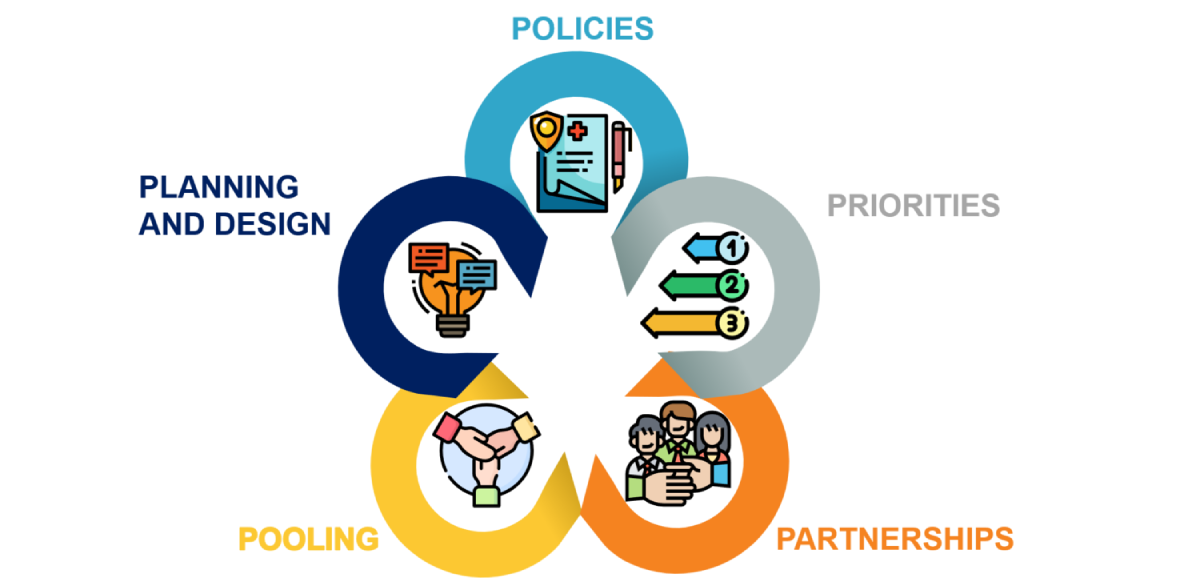
Ingredients to achieve sustained benefits through digital health.

No single best recipe exists, but a list of ingredients may help Italy, and other health care systems worldwide, focus their proposals to achieve sustained benefits (Figure 1):

### Policies

The COVID-19 crisis has reinforced the need to define and uphold appropriate policy standards. Long-standing digital health issues (reimbursement, regulatory framework, evidence generation, privacy, and security concerns) will still be unresolved by the end of this pandemic. Decisive and comprehensive action is thus needed from a policy standpoint, and several extensive proposals have been suggested in other contributions [1,4,18,23,33,35].

### Priorities

The combination of the widespread availability of digital health solutions, their poor uptake before the pandemic, and the confidence gained during the recent expansion may push countries to aim too high. Governments should act promptly, but pragmatically, in adopting a stepped-wedge approach based on their health care system needs and organizational characteristics.

### Partnerships

Digital transformation is bringing in new players with different backgrounds, expertise, and logic compared to the players that have typically populated the health care industry. Public institutions need to welcome innovation and be open to interorganizational relationships and trust. During a public health emergency, occasional, hastily generated partnerships may proliferate. Governments must adopt a strategic approach to partnerships and actively pursue the necessary competencies to harness these opportunities.

### Pooling

The pre-pandemic digital health environment was characterized by a predominance of scattered experiences and the inability to adequately value existing best practices. Higher interinstitutional coordination should be assured by stewardship at the central level to take charge of streamlining the process, identifying parameters and conditions conducive to transferability, and ensuring no one is left behind—all without stifling local innovation.

### Planning and Design

Digital health introduction should be complemented by an in-depth service redesign. The organizational implications of digital health have been largely neglected to date, but they need to be addressed now to exploit the complete potential of digital tools, integrate solutions in current care pathways, and pave the way for new models of care. Any digital technology is only as good as the response it gets from its end-users in terms of acceptability, continuity, and engagement. Hence, new, digitally enriched organizational processes should be planned and designed by all relevant stakeholders, directing patient-provider interactions to the channels generating the most value [36]. Irrespective of the cultural or contract issues, all health care professionals should be involved to steer change processes. Likewise, health care systems should flexibly partner with patients by enacting a mutual learning space and blurring the boundary between producers and consumers [37]. This shared effort at the system level should be pursued in the awareness that digital health literacy, among both citizens and health care professionals, still carries significant weight in influencing the uptake of digital solutions [38]. Although health care systems tackle this issue to grant the benefits of technological advancements to all users, the service redesign should be oriented by process of segmentation that will ideally result in a more tailored response to each individual’s needs through a combination of traditional and digital solutions.

Unprecedented times call for unprecedented decisions. It is time for policymakers to step up and make timely valiant choices about the use of telemedicine and digital health to permanently integrate them into the health care systems for the good of the public health and, ultimately, of the people. This will be extremely helpful now, during the COVID-19 crisis, and even more as we head toward the post-pandemic world.

## Abbreviations

LHA: local health authority
SSN: Italian National Health Service
